# Slow wave canonical activity deviation concept: Toward a slow wave-based EEG-fMRI reference map for health-associated network function

**DOI:** 10.1016/j.isci.2026.115455

**Published:** 2026-03-21

**Authors:** Merve Ilhan-Bayrakcı, Oliver Tüscher, Albrecht Stroh

**Affiliations:** 1 Leibniz Institute for Resilience Research, Mainz, Germany; 2 Department of Psychiatry and Psychotherapy, University Medical Center of the Johannes Gutenberg University Mainz, Mainz, Germany; 3 Institute of Molecular Biology (IMB), Mainz, Germany; 4 Department of Psychiatry, Psychotherapy and Psychosomatics, University Medicine Halle of the Martin Luther University Halle-Wittenberg, Halle (Saale), Germany; 5 German Center for Mental Health (DZPG), Site Halle-Jena-Magdeburg, Halle (Saale), Germany; 6 Institute of Physiology I, University Hospital Muenster, Muenster, Germany

**Keywords:** neuroscience, behavioral neuroscience, sensory neuroscience, cognitive neuroscience, techniques in neuroscience

## Abstract

Functional network architecture associated with health varies across individuals, limiting neuroimaging sensitivity for detecting early network maladaptations and identifying at-risk individuals. To increase sensitivity, we use slow wave events (SWEs) as neurophysiological markers in EEG-informed fMRI analyses. Neural excitability and network state impact SWE spatiotemporal dynamics. Analyzing simultaneous EEG-fMRI from two healthy cohorts (*N* = 24), we generated individual SWE-related BOLD maps. We created cohort-level probabilistic maps and derived reference maps revealing consistently engaged regions, including the cingulate cortex, thalamus, hippocampus, and cerebellum. Stratifying SWEs by synchronization efficiency revealed distinct BOLD patterns. High synchronization SWEs recruited widespread cortical and subcortical networks, whereas low-synchronization SWEs engaged predominantly posterior cortical regions, refining the reference maps for physiologically distinct SWE subtypes. Building on this, we propose the *“slow wave canonical activity deviation (SloCAD)”* concept to quantify individual deviations from canonical SWE activation, providing a foundation for studying early network dysfunction in pathological conditions.

## Highlights

• Reference maps show canonical SWE-related BOLD activation in healthy adults
• Synchronization efficiency-based SWE stratification reveals distinct BOLD patterns
• *SloCAD*: a proposed framework for individual-level deviation from canonical SWE maps

## Graphical abstract

**Figure:**
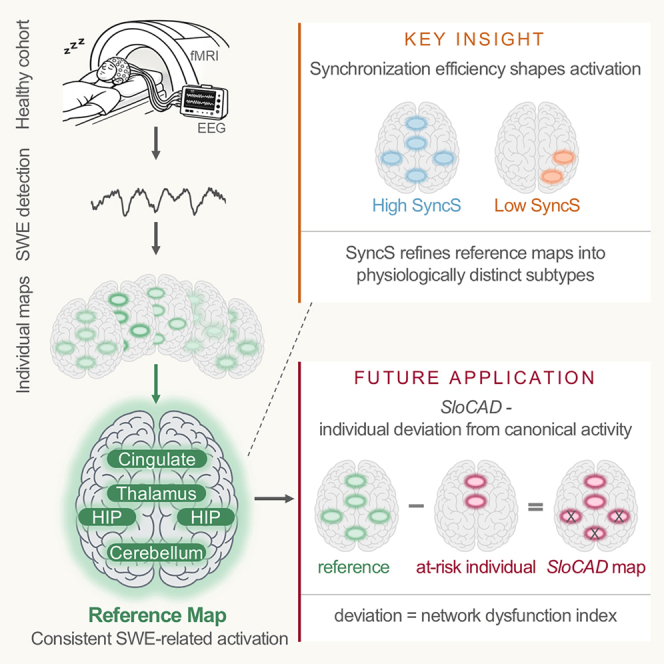

## Introduction

The inherent complexity of the human brain allows its unique cognitive performance, adaptability, and plasticity. This complexity arises from its large number of neurons ranging at 100 × 10^9^.^1^ and their high degree of connectivity.^2^ Since genetic factors only partially predetermine functional connectivity, similar behavioral outputs can emerge from highly variable brain connectivity patterns.

This variability poses a challenge for defining reference maps of healthy brain function. This is of particular relevance for early maladaptive network dysregulations accompanying early stages of neuropsychiatric disorders,^3^ as these early changes might be subtle. Therefore, we are in need of neurophysiological signals that reflect the brain’s functional organizational architecture with high sensitivity. Slow wave events (SWEs), or slow oscillations (SOs), may represent such a neurophysiological event. They provide a window of integration binding remote areas of the brain^4^ and allowing for manifold processes such as memory consolidation.^5^^,^^6^ They are preserved across species^7^^,^^8^^,^^9^^,^^10^^,^^11^ and occur predominantly, but not exclusively, in non-rapid eye movement (NREM) sleep stages N2 and N3. SWEs can be detected non-invasively using scalp electroencephalography (EEG). Analyzing SWEs in EEG recordings combined with functional magnetic resonance imaging (fMRI) enables whole-brain mapping of regions involved in SWE expression at the subject-specific level.^12^

Using night sleep recordings of healthy adults, Dang-Vu et al. conducted group-level analyses and found significant BOLD activation related to SWEs in the pontine tegmentum, midbrain, cerebellum, parahippocampal gyrus, inferior frontal gyrus, middle frontal gyrus, precuneus, and posterior cingulate cortex.^13^ In contrast, Betta et al. investigated SWE-related BOLD signal changes during light sleep in healthy adults with data recorded during an afternoon nap. At group-level analyses, they found SWE-related BOLD activation in the thalamus, brainstem, and cerebellum, along with significant BOLD deactivation in the somatomotor, visual, and insular cortices.^14^ Another recent study by the same group focused on SWE-related BOLD signal changes in school-age children diagnosed with self-limited focal epilepsy of childhood. During an afternoon resting-state scan session where the subjects fell asleep, they observed significant negative hemodynamic changes in the somatomotor cortex but did not detect significant positive changes in subcortical structures during SWEs.^15^ Complementary group-level work by Baena et al. investigated how the temporal coupling between slow waves and spindles modulates the associated BOLD response. They found that uncoupled SWEs activated a recruited region including the hippocampus, thalamus, occipital lobe, postcentral gyrus, superior and inferior temporal gyri, and anterior and posterior cingulate cortices. SWEs coupled with spindles activated a similar network, but additionally recruited the middle frontal gyrus and the putamen.^16^ Lastly, our previous work examined SWE-related BOLD signal changes at the single-subject, single-session level during night sleep, allowing us to capture both interindividual and intraindividual differences. We observed substantial involvement of various parts of the gray matter and structures, including the thalamus, brainstem, and cerebellum, in response to the occurrence of SWEs.^12^

These combined EEG-fMRI studies merging high temporal resolution (EEG) with high spatial resolution (fMRI) lay the groundwork for an SWE-based reference map as a blueprint of health-associated network function. Ideally, such a reference map, derived from a cohort of healthy subjects, must achieve a critical balance: It must characterize how SWEs recruit and link brain regions while accounting for healthy inter-individual variability,^17^^,^^18^ yet remain consistent enough to detect network dysregulations affecting SWE initiation or propagation.^19^

Here, we analyzed individual SWE-related BOLD activations using simultaneous EEG-fMRI recordings from two independent cohorts of healthy adults (total *N* = 24). We constructed voxel-wise engagement probability maps reflecting the likelihood of SWE-related activation across individuals while preserving single-subject resolution, conceptually equivalent to penetrance or overlap maps commonly used to quantify inter-subject consistency and variability in fMRI studies.^20^^,^^21^^,^^22^^,^^23^ In addition, we derived reference maps capturing the regions consistently recruited during SWEs. Together, these maps serve as spatial references for our *slow wave canonical activity deviation concept* (*SloCAD*), which envisions the quantification of individual-specific deviations from healthy cohort-based patterns in clinical contexts.

## Results

### How to create a template of SWE fMRI representation in healthy humans?

To analyze simultaneously recorded fMRI data, we extracted the onset and duration of individually detected SWEs to create vectors encoding their precise timing. These were used to model individual GLMs.

In our previous study, the SWE vector was convolved with the canonical hemodynamic response function (HRF) and its time and dispersion derivatives, with six movement parameters and a constant term included as nuisance regressors. The main effect of SWEs was tested using F-contrasts spanning the canonical HRF and its derivatives, capturing any significant BOLD changes.

**Figure 1:**
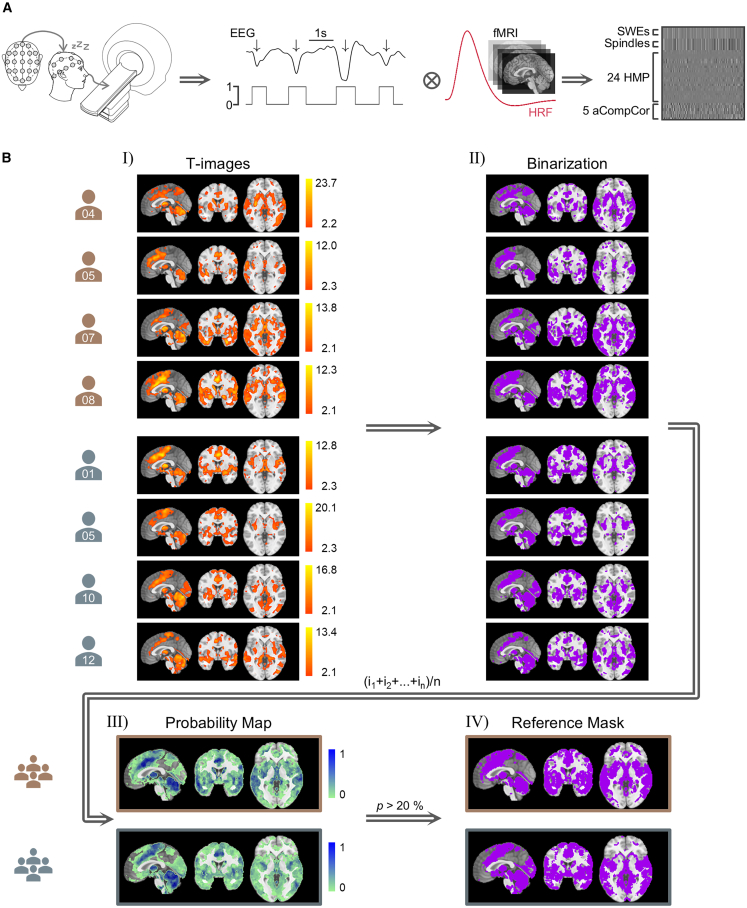
An EEG-informed pipeline with single-subject resolution for generating canonical slow wave event (SWE)-related fMRI activation masks (A) Scheme of simultaneously recorded EEG-fMRI data of sleeping humans and subsequent analysis procedure. SWEs were individually detected in EEG data and converted to binarized event vectors containing the exact onset and duration of each single SWE. For the fMRI analysis, these SWE vectors were convolved with the canonical hemodynamic response function (HRF) and incorporated into a general linear model (GLM) design matrix, along with sleep spindles as regressors of no interest, a set of 24 head motion parameters (HMPs), and five physiological noise regressors extracted from cerebrospinal fluid (CSF) termed anatomical component correction (aCompCor). (B) (Ⅰ) Resulting individual SWE-related BOLD fMRI statistical activation maps, i.e., T-images, thresholded at *q* < 0.05_*FDRcorr*_ with a cluster extent threshold of *k* = 10 voxels. The icons representing individual subjects are colored brown for subjects from dataset 1^27^ and gray for subjects from dataset 2.^28^ (Ⅱ) Conversion of individual T-images to binarized images where activated voxels are coded as ones and non-activated voxels as zeros. (Ⅲ) SWE-related spatial probability masks obtained for each dataset separately by summing all binarized images and dividing them by the respective dataset size. (Ⅳ) Creation of SWE-related spatial reference masks by thresholding the probability masks at a recruitment probability higher than 20%. See Figure S1 for individual-level SWE activation maps.

Here, we implemented an updated model. The primary regressor of interest, SWEs, was convolved with the canonical HRF only. Importantly, we applied a more sensitive EEG detection criterion for SWEs by lowering the amplitude threshold (−5 μV vs. −60 μV in our previous study), thereby enriching the SWE regressor with a broader range of events and enhancing model precision. A secondary regressor for sleep spindles was also convolved with the canonical HRF but included as a regressor of no interest. In addition, we modeled 24 motion parameters (six base parameters - translations and rotations along x, y, and z axes, their derivatives, and quadratic terms),^24^^,^^25^ five CSF-based aCompCor regressors,^26^ and a constant term as nuisance regressors (Figure 1A). The main effect of SWEs was assessed using T-contrasts to identify significant BOLD activations in response to SWEs. These model adjustments substantially improved signal quality and reduced physiological noise, which was particularly important given the long recording durations (range = 60.9–125.6 min).

We applied this modeling approach to two independent datasets of healthy adults recorded during sleep (*N*_*1*_ = 11 and *N*_*2*_ = 13; *N* = 24). The data were originally acquired and published in previous studies by Bergmann et al.^27^ and Sterpenich et al.^28^^,^^29^ For clarity, we refer to them as datasets 1 and 2 throughout the manuscript. Dataset 1 comprises previously unpublished data from the first session (adaptation night) of the Bergmann et al.^27^ study, analyzed in the present study.

This session was not included in our prior work,^12^ which focused exclusively on the two subsequent experimental nights, and therefore represents an independent subset of the cohort. Dataset 2 is publicly available in the OpenNeuro repository^28^ (OpenNeuro: ds003574, DOI: https://openneuro.org/datasets/ds003574/versions/1.0.2).

Consistent significant BOLD activations across individuals were observed in key regions, including the cingulate gyrus, thalamus, hippocampus, and cerebellum (Figures 1B and S1). These patterns reflect the long-range coupling of activity between the cortex, thalamus, and hippocampus, highlighting a key target for studying SWE alterations.

To generate probabilistic templates of SWE-related activation, each subject’s thresholded T-map (*q* < 0.05_*FDRcorr*_, *k* = 10 voxels) was binarized. Summing these and dividing by sample size yielded voxel-wise probability maps, generated separately for each dataset. Both maps showed high overlap in SWE-recruited regions, notably the cingulate gyrus, thalamus, hippocampus, and cerebellum (Figure 1B).

The probability maps represent one outcome of our SWE-informed fMRI analysis pipeline, providing insight into both consistent and variable engagement of brain regions across individuals. To create binary reference masks from the probabilistic maps, we applied a threshold of 20% probability for an SWE recruiting a specific voxel, ensuring sensitivity to interindividual variability while maintaining a degree of specificity. Setting this threshold will depend on 1) the sample size and 2) the homogeneity of the subjects in terms of their age, sex, and physiological state. This balances the need for a robust mask and the necessity to include the intrinsic physiological variability of SWEs.

The resulting reference masks comprised 74300 voxels for dataset 1 and 80826 voxels for dataset 2 (Figure 1B). The masks shared 52944 voxels in common, representing 71.3% of the mask from dataset 1 and 65.5% of the mask from dataset 2. This substantial overlap reflects consistent patterns of SWE-related activation across independent cohorts. Shared cortical regions included the cingulate gyrus, medial frontal gyrus, paracentral lobule, insula, and temporal gyri (superior, middle, inferior), as well as the parahippocampal gyrus. Subcortically, overlap was observed in the thalamus, hippocampus, amygdala, caudate, putamen, cerebellum, and brainstem. The reference masks are publicly available via OSF (OSF: 6adf2; DOI: https://doi.org/10.17605/OSF.IO/6ADF2) at https://osf.io/6adf2/.

### Do SWEs with putatively differing neurophysiological roles result in distinct spatial reference masks?

**Figure 2:**
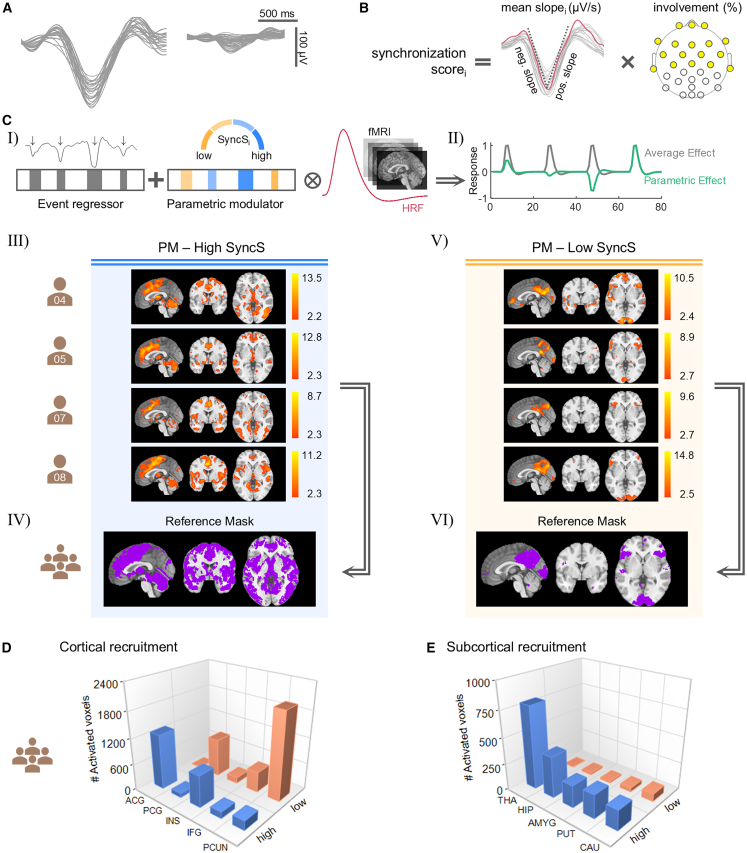
Slow wave events (SWEs) with higher versus lower synchronization efficiency are associated with distinct BOLD patterns (A) Examples of SWEs demonstrate substantial variability in key characteristics such as amplitude, slope, and duration. (B) Calculation scheme of the synchronization score (SyncS) for each detected SWE, following the methodology of Bernardi et al.^30^ This score reflects the synchronization efficiency of SWEs and is calculated by multiplying the average of the negative and positive slopes by the relative scalp involvement, which represents the percentage of channels contributing to the SWE within a 40 ms time window. (C) (Ⅰ) Incorporation of synchronization scores into fMRI analysis. Event regressors containing the precise timing and duration of SWEs, combined with parametric modulation regressors that include synchronization scores for each SWE, were convolved with the canonical HRF. (Ⅱ) The parametric effect reflects how BOLD response amplitude varies with the magnitude of the synchronization score. (Ⅲ and Ⅳ) BOLD response patterns of individual subjects show brain regions where the BOLD signal increases with higher SyncS *(q* < 0.05_*FDRcorr*_*,* cluster extent threshold: *k* = 10 voxels*)*, along with the corresponding spatial reference mask. (Ⅴ and Ⅵ) BOLD response patterns of individual subjects highlighting brain regions where the BOLD signal increases with lower SyncS *(q* < 0.05_*FDRcorr*_*,* cluster extent threshold: *k* = 10 voxels*)*, along with the corresponding spatial reference mask. (D) 3D bar plot shows mean activated voxel counts across cortical regions for SWEs with higher (blue) and lower (orange) SyncS. Data are represented as the mean across subjects. Abbreviations: ACG, anterior cingulate gyrus; PCG, posterior cingulate gyrus; INS, insula; IFG, inferior frontal gyrus; PCUN, precuneus. (E) 3D bar plot shows mean voxel recruitment in subcortical regions for SWEs with higher (blue) and lower (orange) SyncS. Data are represented as the mean across subjects. Abbreviations: THA, thalamus; HIP, hippocampus; AMYG, amygdala; PUT, putamen; CAU, caudate. See also Figures S2 and S3.

SWEs vary in both temporal dynamics and regional recruitment. While variability is inherent, further classification may reveal functionally distinct subtypes. Following Bernardi et al.,^30^ we characterized SWEs using the synchronization score (SyncS), a continuous measure reflecting both the extent and speed of neural recruitment and indexing synchronization efficiency (Figure 2B).

To examine how synchronization efficiency modulates BOLD responses, we included a parametric modulation regressor in the GLMs encoding the SyncS of each SWE (Figure 2C). This approach allowed us to identify brain regions in which BOLD responses systematically varied with synchronization efficiency. Subject-level examples from dataset 1 are shown in Figure 2C, with full results provided in Figure S2 (dataset 1) and Figure S3 (dataset 2).

This analysis yielded two reference masks for each dataset, one representing SWEs with higher SyncS and one representing SWEs with lower SyncS. For dataset 1, the mask for SWEs with higher SyncS included 64833 voxels (Figure 2C), and for dataset 2, 57286 voxels (Figure S3A). SWEs with higher SyncS exhibited robust activation in the anterior cingulate, medial frontal gyrus, paracentral lobule, insula, superior, middle, and inferior temporal gyri, parahippocampal gyrus, and subcortical regions including the thalamus, hippocampus, amygdala, caudate, putamen, and cerebellum.

In contrast, SWEs with lower SyncS predominantly activated the posterior cingulate, inferior frontal gyrus, precuneus, cuneus, and lingual gyrus, with comparatively reduced subcortical involvement. The masks for SWEs with lower SyncS included 19168 voxels (dataset 1; Figure 2C) and 39848 voxels (dataset 2; Figure S3A).

To assess whether these spatial patterns were influenced by SWE-spindle coupling, we conducted a follow-up analysis in which each SWE was classified as coupled or uncoupled. The BOLD patterns related to SWEs with higher and lower SyncS were then examined after accounting for SWE-spindle coupling. The resulting spatial patterns were largely unchanged (see Figures S4 and S5), indicating that the SyncS reflects intrinsic differences in synchronization efficiency rather than an effect of SWE-spindle coupling.

### Do sleep stages impact the spatial representation of SWEs?

SWEs are a hallmark of NREM stages N2 and N3, though they can also occur during wakefulness.^31^^,^^32^^,^^33^^,^^34^ Given the distinct neuromodulatory tones of sleep stages,^35^ we examined whether SWE-related BOLD responses differ between N2 and N3, as these two stages are hypothesized to serve distinct functions,^36^ and differentiation between these stages is common practice in existing literature.^37^^,^^38^

**Figure 3:**
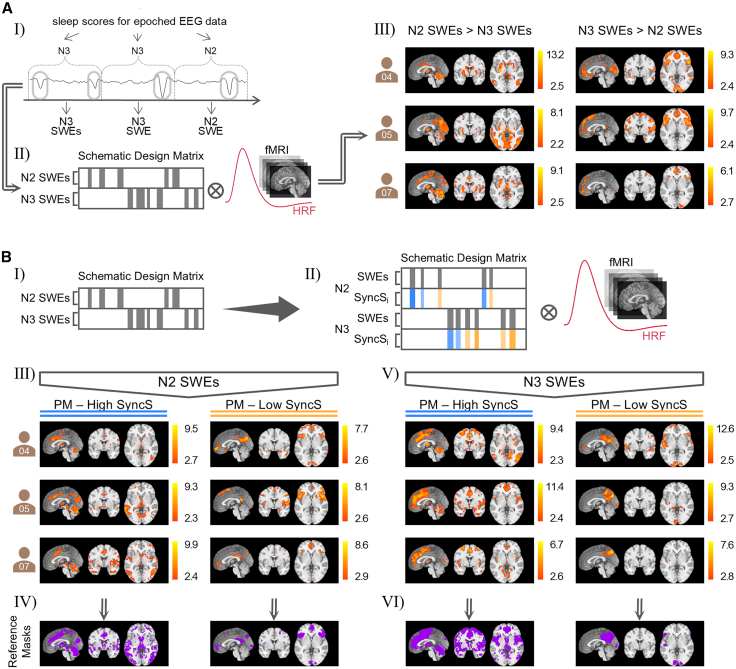
Synchronization efficiency, rather than sleep stages, determines slow wave event (SWE)-related BOLD activation patterns (A) (Ⅰ) Scheme of sleep-scored EEG data showing how SWEs are classified according to the sleep stage in which they occurred. (Ⅱ) Schematic design matrix including two event regressors: one for SWEs during sleep stage N2 and one for SWEs during sleep stage N3. Both regressors were convolved with the canonical HRF. (Ⅲ) Subject-wise results images depicting regions that showed stronger BOLD activation in response to N2 SWEs compared to N3 SWEs and vice versa *(q* < 0.05_*FDRcorr*_*,* cluster extent threshold: *k* = 10 voxels*)*. (B) (Ⅰ) Design matrix for contrasting N2 versus N3 SWEs. (Ⅱ) Parametric modulators encoding synchronization scores (SyncS) were added to test interactions with sleep stage. Event regressors and parametric modulators were convolved with the canonical HRF. (Ⅲ) Individual BOLD responses for N2 SWEs: The left column highlights brain regions where the BOLD signal increases with higher SyncS, while the right column highlights brain regions where the BOLD signal increases with lower SyncS *(q* < 0.05_*FDRcorr*_*,* cluster extent threshold: *k* = 10 voxels*)*. (Ⅵ) Corresponding spatial reference masks for each column. (Ⅴ) Individual BOLD responses for N3 SWEs: The left column shows brain regions where the BOLD signal increases with higher SyncS, and the right column shows brain regions where the BOLD signal increases with lower SyncS *(q* < 0.05_*FDRcorr*_*,* cluster extent threshold: *k* = 10 voxels*)*. (Ⅵ) Corresponding spatial reference masks for each column.

We modeled two separate event regressors for fMRI analyses: one for SWEs during N2 and one for SWEs during N3 (Figure 3A). We examined the regions that were significantly more activated by N2 SWEs as compared to N3 SWEs and the regions that were significantly more activated by N3 SWEs as compared to N2 SWEs, and vice versa. The BOLD activation maps of N2 and N3 SWEs displayed variations in their spatial recruitment patterns. N2 SWEs exhibited stronger activation in the anterior cingulate gyrus, thalamus, hippocampus, and cerebellum, while N3 SWEs showed greater activation in areas including the frontal gyri (superior, middle, inferior) and posterior cingulate gyrus.

Notably, the spatial profiles of N2 SWEs resembled those of SWEs with higher SyncS, whereas N3 SWEs resembled SWEs with lower SyncS. To determine whether sleep stage or synchronization efficiency primarily shapes the spatial representation of SWEs, we included parametric modulators of SyncS within each stage (N2 and N3).

Comparisons of SWEs with higher SyncS across N2 and N3 revealed a high degree of similarity, and the same was observed for SWEs with lower SyncS (Figure 3B). These results indicate that synchronization efficiency, rather than sleep stage, at least not N2- or N3-stages, predominantly determines the BOLD representation of SWEs.

### The slow wave canonical activity deviation concept: SloCAD

In the previous sections, we explored concepts of refining an SWE-based reference map and found that incorporating synchronization efficiency provides a valuable improvement over undifferentiated approaches. Despite relatively small sample sizes (*N*_*1*_ = 11 and *N*_*2*_ = 13), both datasets yielded substantially overlapping reference masks, suggesting that this conceptual pipeline could bear early diagnostic value. This is particularly promising given key design differences between the two studies, such as sleep timing, pre-sleep tasks, and sleep deprivation.

**Figure 4:**
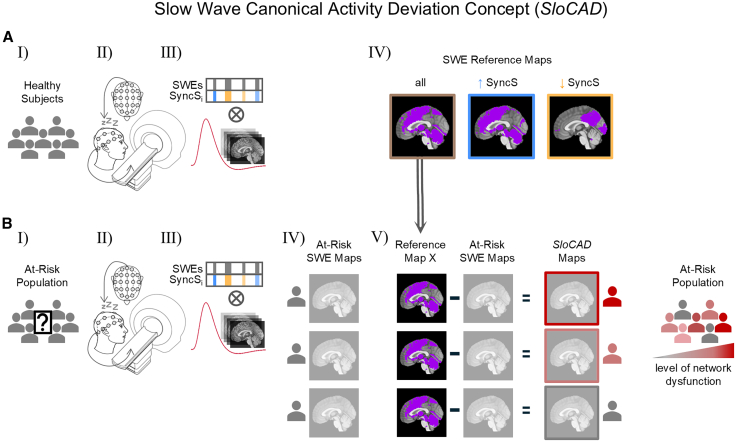
Slow wave canonical activity deviation concept (SloCAD) (A) (Ⅰ) Selection of a healthy subject sample. (Ⅱ) Participants are subjected to a sleep EEG-fMRI study protocol. (Ⅲ) SWEs detected in the EEG data and their synchronization scores (SyncS) are included in event-related fMRI analyses as described. (Ⅳ) Creation of SWE-related differential spatial reference masks. (B) (Ⅰ) Selection of an at-risk population. (Ⅱ) Participants are subjected to a sleep EEG-fMRI study protocol. (Ⅲ) SWEs detected in the EEG data and their synchronization scores are included in event-related fMRI analyses. (Ⅳ) Extraction of subject-wise SWE-related BOLD activation maps. (Ⅴ) Subtraction of individual SWE-related BOLD activation maps from a reference map of interest, which leads to SloCAD maps, enabling the study of an individual’s extent of deviation from the canonical activity represented in the reference mask. The extent of deviation might reflect the level of network dysfunction.

Notwithstanding other meaningful classifications of SWEs at this point, we would propose that this pipeline could be applied to a larger study yielding three distinct reference masks (Figure 4A). We present “*SloCAD.”* Though the SyncS was used here to differentiate SWEs based on their synchronization efficiency, other neurophysiologically grounded classification schemes could explain more variance in slow wave activity (SWA) across healthy individuals.

*SloCAD* involves several key steps: First, establish a population-based cohort of healthy controls, age-matched controls, given the strong of age on SWA.^39^ This cohort undergoes combined EEG-fMRI during sleep, ideally in N2 and N3 stages to maximize SWE detection. SWE-related BOLD maps are then generated to create subtype-specific reference masks. (Figure 4A).

The age-matched at-risk population is being identified due to specific genetic, environmental, or socioeconomic vulnerabilities toward a disease entity. The identical EEG-fMRI protocol and analysis scheme as conducted for the healthy subjects will be performed. Again, subject-wise SWE-related activation maps are generated, which are next subtracted from a previously generated healthy SWE reference map of interest. This results in subject-specific *SloCAD* maps showing the deviation from the canonical reference map. In addition, a quantitative metric of the overall deviation is yielded. This approach allows each at-risk individual to be assigned a deviation metric, which we hypothesize reflects the level of (early) network dysfunction.

## Discussion

### Distinct regional recruitment patterns of SWEs

SWEs support various brain functions, including memory consolidation,^5^ synaptic downscaling,^40^ and the facilitation of metabolic waste removal.^41^^,^^42^^,^^43^ Given these diverse roles, it is plausible that SWEs engage different brain regions and serve distinct functional purposes.

SWEs have been classified based on their spatiotemporal properties, synchronization efficiency, and thalamocortical involvement. Malerba et al.^37^ stratified SWEs into global, local, and frontal types based on their spatiotemporal characteristics, demonstrating that global SWEs have higher amplitudes, stronger spindle coupling, and fronto-posterior traveling profiles compared to the other two classes. Seok et al.^38^ used machine learning on depth profiles and source localization to differentiate global from non-global SWEs. Siclari et al.^44^ distinguished two types of SWEs based on synchronization efficiency: Type I SWEs, characterized by high amplitude, steep slopes, and widespread cortical activation, likely driven by thalamocortical activations; and Type II SWEs, with lower amplitude, flatter slopes, and more localized cortical involvement, indicative of cortico-cortical connections. Bernardi et al.^30^ quantified this distinction by introducing a SyncS, integrating spatial spread (relative scalp involvement—percentage of channels contributing to an SWE) and synchronization speed (mean slope). Building on this, Navarrete et al.^45^ identified three SWE categories based on synchronization efficiency: one resembling Type I waves, another aligned with Type II waves,^30^^,^^44^ and a third distinct category that shares features of both types I and II waves, combining widespread spatial distribution with lower amplitudes and flatter slopes.

Bergamo et al.^46^ used fMRI-based clustering to classify SWEs into two types based on their thalamic BOLD response profiles. They identified a C1 cluster, characterized by large, highly synchronized waves linked to early thalamic activation, and a C2 cluster, consisting of smaller, more localized waves with delayed thalamic responses, primarily occurring during stable sleep. These findings support the existence of SWE subtypes with distinct spatial profiles and thalamocortical dynamics, which Bergamo et al.^46^ suggest may be differentially affected in neurological or psychiatric conditions. This highlights the need to resolve differential BOLD patterns associated with each SWE type.

In the present work, the incorporation of synchronization efficiency into our event-related fMRI analysis revealed distinct BOLD patterns for high versus low synchronization SWEs, highlighting the utility of this metric for characterizing SWE-related brain activity. Previous work has shown that specific types of SWEs, particularly those with broader spatial recruitment and steeper slopes, tend to be more strongly coupled to spindles.^37^^,^^45^ To determine whether the differential BOLD patterns related to high versus low SyncS SWEs were influenced by SWE-spindle coupling, we performed a follow-up analysis in which SWE-spindle coupling was accounted for. The BOLD patterns associated with high and low SyncS remained largely unchanged, indicating that SyncSs capture intrinsic differences in synchronization efficiency rather than an effect of SWE-spindle coupling. This interpretation aligns with Baena et al.,^16^ who reported only limited BOLD fMRI differences between coupled and uncoupled SWEs, and whose conjunction analysis showed that both event types engage a largely overlapping core network, in contrast to the clearly differentiated BOLD patterns corresponding to high- and low-synchronization SWEs in our dataset.

While the high versus low SyncS BOLD patterns diverged, one central region consistently played a key role in both types: the cingulate gyrus. Its activation, which was not a consistent finding in previous studies examining SWE-related BOLD activation, was prominently observed across both datasets of the present study. Our findings align with Murphy et al.,^9^ who used high-density EEG to show that SWEs prominently engage the cingulate gyrus. Notably, the cingulate gyrus serves not only as a hotspot for SWE initiation but also as a central hub through which SWEs from various origins propagate.^9^ Results from functional neuroimaging studies of animal models of neuropsychiatric disorders suggest that the cortex is particularly vulnerable to early network changes,^47^^,^^48^^,^^49^ which can critically affect SWE propagation.^19^ Recent methodological advances in rodents, such as fast line-scanning fMRI combined with optical recordings, make it possible to resolve the continuous cortical trajectory of individual SWEs.^50^ Once adapted to human studies, these approaches could enable targeted investigation of hub regions such as the cingulate gyrus, which may play a critical role in altered SWE dynamics and disrupted propagation, alongside other relevant regions.

### SWEs: Ideal markers for neuropsychiatric brain (Dys-) function

We follow the concept that network dysregulation occurs early in the disease course, prior to the onset of any disease-specific clinical symptoms. Moreover, these network dysregulations may not only be temporally but also spatially dissociated from the region where the primary neuropathophysiological event occurs.^3^ Consequently, it is essential to develop a reference map of healthy network function that is not restricted to a specific brain region, which would entail the risk of missing dysregulated hubs.

An SWE-related reference mask, therefore, might be able to fit these requirements as SWEs serve for a long-range integration necessary for, e.g., memory consolidation^5^ and recruit widespread cortical and subcortical regions. For an SWE-based reference map to serve as an early sign of network deviation toward disease, it must explain a significant fraction of the variance. This is particularly important because, based on our previous study, there appears to be high inter and intra-individual variability in the representation of SWEs.^12^ Therefore, we need to design a map that captures this variability, fulfilling the critical need to model what constitutes “normal/typical” in controls before characterizing the atypical,^17^^,^^18^ while remaining sensitive to pathophysiological maladaptation.

### Implications of SloCAD in early network dysfunction

Unifying the approach of SWE-based reference maps and the putative sensitivity of SWEs toward early network dysregulations, we propose an open concept for assessing early network dysfunction in an inherently variable functional connectome. Focusing on SWEs and even characterizing them in functionally distinct subtypes may justify proposing the *“SloCAD.”* It provides a conceptual framework of distinct steps that can be adopted for a given cohort and a given disease entity. It is likely to assume that SWE recruitment and propagation should be affected in a multitude of neuropsychiatric disorders, even though the molecular pathophysiology and the regions affected first might greatly differ. We propose to consider the disease-specific regional preference of the first occurrence of network dysregulations. In the case of Alzheimer’s disease (AD), this could entail the hippocampus and the entorhinal cortical areas,^51^^,^^52^ in the case of Huntington’s disease (HD), it could be the striatum.^53^ In the case of Parkinson’s disease (PD), it could be the basal ganglia or olfactory circuits.^54^^,^^55^ This could entail assigning different weights to a given region. However, the strength of *SloCAD* relies on the fact that, despite putative weighting, we consider the entire brain and are still sensitive toward non-canonical deviations.

As an open concept, *SloCAD* invites the introduction of other neurophysiological events which may represent functional integrity, such as sleep spindles, K-complex, and sharp wave ripples. The essence of *SloCAD* can be summarized as achieving a single subject-specific activation map of a defined neurophysiological event and contrasting this activation map with the canonical functional architecture represented in a reference map.

Beyond its diagnostic potential, *SloCAD* has implications for therapeutic intervention. Understanding network dysfunction through SWE alterations can inform the development of neuromodulatory and pharmacological strategies. For instance, closed-loop transcranial alternating current stimulation (CL-tACS) targeting SWEs during sleep has emerged as a promising tool to enhance cognitive and restorative processes. Robinson et al.^56^ demonstrated improvements in sleep quality and efficiency, particularly following nights of disrupted sleep. Similarly, Ketz et al.^57^ revealed that SWE-synchronized CL-tACS enhances memory generalization by boosting SWE/spindle coupling, while Jones et al.^58^ showed that CL-tACS can reduce retroactive interference, particularly in individuals with weaker memory encoding. A central challenge, however, is the precise identification of which SWEs to target for maximal therapeutic efficacy, particularly in diseased brains.

Our findings highlight that not all SWEs are equivalent, a distinction with direct therapeutic relevance. Early network dysregulations, such as local hyperexcitability in the early stages of AD,^49^ HD,^47^ and MS,^48^ may impair the long-range propagation of SWEs. Consistent with this, Busche et al.^19^ showed disrupted SWE propagation, reflecting a breakdown of large-scale functional connectivity among cortex, hippocampus, and thalamus. In our data, only high-synchronization SWEs recruit this thalamocortical-hippocampal network, whereas low-synchronization SWEs remain confined to the cortex. Thus, high-synchronization SWEs represent the SWE subtype probably most vulnerable to early-stage network dysregulations.

This selective vulnerability also makes high-synchronization SWEs a promising target for therapeutic interventions. Defined by specific slope-and-involvement metrics, they are not a novel construct but correspond directly to established classifications of globally propagating SWEs, namely Bernardi’s widespread, high-amplitude Type I waves and Navarrete’s C1 cluster of global, steep-slope waves.^30^^,^^45^ Critically, this subtype is not just observable but also targetable: Closed-loop acoustic stimulation has been shown to selectively enhance it,^45^ and recent approaches allow for the precise optimization of stimulation parameters to target them effectively.^59^ Promoting high-synchronization SWEs may therefore counteract early propagation deficits and restore network communication.

Pharmacological interventions further emphasize the importance of early, targeted treatment. For example, Kollarik et al.^60^ found that sodium oxybate improved cognition and reduced pathology in an Alzheimer’s mouse model when administered early, whereas later treatment failed to rescue deficits. Collectively, these findings position high-synchronization SWEs as a propagation-sensitive event class, providing a concrete target for early detection and intervention in neuropsychiatric disorders.

### Future directions and opportunities for SloCAD

The substantial spatial convergence of reference maps across two independent datasets (*N*_*1*_ = 11 and *N*_*2*_ = 13) indicates consistent and biologically plausible neurophysiological patterns, detected even at rather small sample sizes.

Establishing biological and clinical relevance will require large multi-center studies integrating behavioral or cognitive measures and including both healthy and patient cohorts. Future work should employ standardized EEG-fMRI acquisition protocols and may incorporate bootstrapping strategies to quantify sample-size stability,^61^ alongside computational recruitment strategies to guide multi-site data collection toward optimal cohort representativeness.^62^ As datasets expand, for example, through ENIGMA-Sleep consortium^63^ including multimodal recordings, the *SloCAD* pipeline can be validated against clinical outcomes, determining whether individual SWE patterns serve as biomarkers of network health.

### Limitations of the study

The present study should be considered a methodological proof of concept: Although *SloCAD* provides a structured and reproducible framework, it has not yet been validated as a clinical marker, and the reference maps derived here do not constitute a population-level normative atlas. While numerous EEG studies report SWA alterations in neuropsychiatric conditions such as AD,^64^ PD,^65^^,^^66^ and schizophrenia,^67^ simultaneous sleep EEG-fMRI acquisitions in these populations are absent. Sleep EEG-fMRI has been applied in epilepsy^68^^,^^69^^,^^70^ and insomnia,^71^^,^^72^^,^^73^ but for neuropsychiatric cohorts such as AD and PD, only resting-state EEG-fMRI studies in the awake exist up to now.^74^^,^^75^^,^^76^ Together, these limitations prevented us from testing the framework on patient data. Technical challenges, including scanner noise, movement restrictions, and extended recording durations, contribute to small sample sizes and high dropout rates,^77^ constraining generalizability. Major repositories reflect this gap: The National Sleep Research Resource includes no sleep EEG-fMRI datasets despite over 27 sleep studies,^78^ the ENIGMA-Sleep consortium (>20,000 individuals) lacks this modality,^63^ and OpenNeuro.org hosts only three modest healthy cohorts (*N* = 16–33).

## Resource availability

### Lead contact

Requests for further information, resources, and reagents should be directed to and will be fulfilled by the lead contact, Merve Ilhan-Bayrakci (merve.ilhan@lir-mainz.de).

### Materials availability

This study did not generate new unique reagents.

### Data and code availability

• **Data:** The Bergmann et al.^27^ data (dataset 1) reported in this study cannot be deposited in a public repository because they are subject to ethical restrictions. To request access, contact the Ethics Committee of the Medical Faculty, Christian-Albrechts-University of Kiel (Project: *“Schlafbedingte Gedächtniskonsolidierung assoziativen Lernens: eine kombinierte fMRT EEG Studie,”* Reference A 108/08; ethikkomm@email.uni-kiel.de). This paper analyzes existing, publicly available data (dataset 2) available via OpenNeuro (OpenNeuro: ds003574; DOI: https://openneuro.org/datasets/ds003574/versions/1.0.2).^28^^,^^29^ Reference masks generated in this study have been deposited in OSF (OSF: 6adf2; DOI: https://doi.org/10.17605/OSF.IO/6ADF2) and are publicly available at https://osf.io/6adf2/.
• **Code:** This paper does not report original code.
• **Additional information:** Any additional information required to reanalyze the data reported in this paper is available from the lead contact upon request.

## Acknowledgments

We thank the Leibniz Association (SAW Learning Resilience, LRA-RA LFV-2021-2-LIR), the 10.13039/501100008454Boehringer Ingelheim Foundation, the State of Rhineland-Palatinate (ReALity Innovation Fund P8 and P9), the 10.13039/501100000780European Union (H2020-SC1-2017-CNECT-2: grant no. 777084 DynaMORE), the 10.13039/100000002National Institutes of Health (PO1 HL14454 (Project 2), R01 HL144801, R01 HL151389, and R01 HL126523), the 10.13039/501100002241Japan Science and Technology Agency (Moonshot R&D Goal 9 JPMJMS2292-1-05), and the 10.13039/501100001659German Research Foundation (TRR 128, SFB1193, and SFB1080) for their support.

## Author contributions

Conceptualization, M.I-B., O.T., and A.S.; methodology, M.I-B.; formal analysis, M.I-B.; visualization, M.I-B.; writing – original draft, M.I-B., O.T., and A.S.; writing – review and editing, M.I-B., O.T., and A.S.; supervision, O.T. and A.S. All authors read and approved the final manuscript.

## Declaration of interests

The authors declare no competing interests.

## STAR★Methods

### Key resources table

**Table:** 

REAGENT or RESOURCE	SOURCE	IDENTIFIER
**Deposited data**		

Raw data of dataset 2	OpenNeuro repository	https://openneuro.org/datasets/ds003574/versions/1.0.1https://openneuro.org/datasets/ds003574/versions/1.0.2
Reference maps	This paper; OSF repository	https://osf.io/6adf2/?view_only=4e8ddcc68e294d26a64ce7a0bdcafc79 OSF: 6adf2; DOI: 10.17605/OSF.IO/6ADF2; https://osf.io/6adf2/.

**Software and algorithms**		

MATLAB_R2021b	Math Works, Natick, MA, USA	https://www.mathworks.com/products/matlab.html
EEGLAB v2023.1	Swartz Center for Computational Neuroscience, UC San Diego	https://sccn.ucsd.edu/eeglab/index.php
Sleep Wave Analysis toolbox (swa-matlab)	Mensen et al.^79^	https://github.com/Mensen/swa-matlab
The Bergen fMRI Toolbox Plugin for EEGLab	Moosmann et al.^80^	https://sccn.ucsd.edu/eeglab/plugin_uploader/plugin_list_all.php
The PREP pipeline	Bigdely-Shamlo et al.^81^	https://github.com/VisLab/EEG-Clean-Tools
TESA software	Rogasch et al.^82^; Mutanen et al.^83^	https://nigelrogasch.github.io/TESA/
ICLabel	Pion-Tonachini et al.^84^	https://iclabel.ucsd.edu/
Yet Another Spindle Algorithm (YASA)	Vallat and Walker^85^	https://yasa-sleep.org/index.html
HALFpipe (Harmonized AnaLysis of Functional MRI pipeline) version 1.2.2	The ENIGMA Consortium	https://github.com/HALFpipe/HALFpipe
Statistical Parametric Mapping (SPM 12)	Wellcome Center for Human Neuroimaging, UCL	https://www.fil.ion.ucl.ac.uk/spm/software/spm12/;

### Experimental model and study participant details

#### Origin and characteristics of datasets

This study reanalyzed previously collected human EEG-fMRI datasets. No new participants were recruited, and no new data was collected for the present study.

Dataset 1 is a subset of a previously published study investigating sleep spindle-related reactivation of category-specific cortical regions after learning face–scene associations,^27^ comprising *N* = 11 subjects (mean age: 24.5 ± 1.9; age range = 21–27; 4 females, 7 males). The dataset includes three separate EEG-fMRI recording sessions per subject. Both the original publication^27^ and our previous study^12^ focused exclusively on the second and third sessions, corresponding to the two experimental nights. In the present study, we exclusively use data from the previously unpublished first session (adaptation night), analyzed in the present study. This session was not included in any prior publication and therefore represents an independent subset of this cohort. Participants had no history of neurological or psychiatric disease and were not on medication. They were instructed to avoid alcohol and caffeine the day before and to restrict sleep to 4 h the preceding night. Sleep recordings began around 11 p.m. and lasted up to 2.5 h fMRI data were acquired using a 3T Philips Achieva scanner, and EEG with a 32-channel MR-compatible cap (BrainCap MR, Easy-Cap, Munich, Germany).

Dataset 2 is from an open-access EEG-fMRI dataset on OpenNeuro (OpenNeuro: ds003574),^28^ originally investigating reward-related spontaneous neural reactivations during sleep.^29^ It includes *N* = 18 subjects, but we focused on *N* = 13 subjects (mean age: 22.0 ± 2.5; age range = 18–26; 9 females, 4 males) for consistency with prior work.^29^ Participants had no history of neurological or psychiatric disease and were not on medication. They were instructed to maintain a regular sleep schedule. Recordings started around 10 p.m. and lasted up to 2 h and 40 min fMRI data were acquired using a 3T Siemens Tim Trio scanner, and EEG with a 64-channel MR-compatible cap.

All procedures in the original studies were approved by the respective institutional ethics committees (Ethics Committee of the University of Kiel, Germany^27^; Human Research Ethics Committee, State of Geneva, Switzerland^28^^,^^29^), and written informed consent was obtained from all participants prior to participation. Full protocol and acquisition details are described in the original publications. No additional demographic information (e.g., gender identity, ancestry, race/ethnicity, or socioeconomic status) was available. Given the small sample sizes, analyses of sex differences were not performed.

### Method details

#### EEG preprocessing

The EEG data were preprocessed in MATLAB (The MathWorks, Inc., version 2021b.) using EEGLAB v2023.1.^79^ Scanner gradient artifacts were removed using the ‘realignment parameter-informed’ algorithm from the BERGEN toolbox,^80^ applied only to dataset 1, as dataset 2 was already free of scanner gradient artifacts. From this point onward, identical preprocessing steps were applied to both datasets. EEG data were downsampled to 200 Hz. Preprocessing closely followed the Alesandrelli et al. protocol.^81^ Using the EEGLAB PREP pipeline^82^ we detrended EEG signals via a 1.5 s sliding window with 0.02 s steps, and detected bad channels based on amplitude, SNR, inter-channel correlation, and RANSAC predictions. These were temporarily excluded. Next, a 50 Hz notch and 0.5–45 Hz Butterworth bandpass filter were applied via the EEGLAB TESA plugin.^83^ Independent component analysis (ICA) identified ocular, muscular, and electrocardiographic artifacts. Independent components (ICs) were classified using ICLabel,^84^ and artifactual ICs were removed. Excluded channels were restored via spherical spline interpolation, and data re-referenced to linked mastoids (TP9, TP10).

#### SWE detection

SWEs were detected using the Sleep Wave Analysis toolbox (swa-matlab) by Mensen et al.^85^ For SWE detection, it includes an algorithm that calculates the negative envelope of all electrodes which is used as a reference signal for subsequent SWE detection. Specifically, it involves selecting the four most negative samples at each time point (across electrodes) from the 0.5–4 Hz filtered signal, discarding the single most negative sample, and averaging the remaining three values. SWEs were detected on the baseline-corrected (zero-mean centered) negative signal envelope by applying a procedure based on the detection of negative half-waves.^86^ Half-waves lasting 0.25–1 s were classified as SWEs. An amplitude threshold of −5 μV was applied to reduce noise, following Navarrete et al.^45^ Onsets (first zero-crossing) and durations (positive-to-negative to negative-to-positive zero-crossing) were extracted to create event vectors for fMRI analysis.

Key SWE characteristics were extracted as in Bernardi et al.^30^ The negative slope (μV/s) was determined by calculating the gradient between the wave’s onset and its maximum negative peak. The positive slope (μV/s) was calculated from the wave’s maximum negative peak to the point where the waveform becomes positive. To quantify the number of channels contributing to an SWE, the relative scalp involvement was calculated as the percentage of channels with a negative average current value below −5 μV within a 40 ms time window centered on the maximum negative peak. With these three features, the synchronization score, as originally defined by Bernardi et al.,^30^ was calculated using the relative scalp involvement multiplied by the SWE mean slope (the average of the negative and positive slopes). However, for the sake of reproducibility, we have adopted a specific equation from a recent preprint^81^:

$$synchronization\;score=involvement\times \frac{positive\;slope\;+\;negative\;slope}{2}\times \frac{1}{1000}$$

#### Spindle detection

Spindles were detected from channel Cz using the swa-matlab.^85^ The EEG signal was filtered in the 10–16 Hz range with a b-spline wavelet-based filter. Power time series were computed by squaring values and smoothing with a 100 ms sliding window. Potential spindles were identified when power values surpassed a high threshold set at the median plus four times the median absolute deviation (MAD) of signal power. Onset and offset were defined by crossings at a lower threshold, defined as the median power plus two MADs. Thresholds were updated for each 30-s epoch. Events lasting 0.3–3 s were retained. To ensure specificity, a power-ratio criterion was applied, comparing the mean power in the spindle range (10–16 Hz) to the mean power in adjacent frequency ranges (8–10 Hz and 16–18 Hz), with only those events showing a ratio greater than 3 being retained.

#### Slow wave–spindle coupling analysis

Each slow wave event (SWE) was classified as coupled or uncoupled based on the temporal relationship between the SWE negative peak and the spindle peak. A 4-s time window was defined around the SWE negative peak, following Baena et al.^16^ An SWE was classified as coupled if a spindle peak occurred within this window and uncoupled otherwise.

#### Sleep scoring

EEG of dataset 1 were sleep scored using *Yet Another Spindle Algorithm (YASA)*.^87^ Sleep stages (wake, N1, N2, N3, or REM sleep) were determined in each 30-s epoch. Channel C3 (re-referenced to averaged mastoids) served as input, along with EMG and VEOG signals.

For dataset 2, sleep scoring by the original authors was provided via OpenNeuro (ds003574).^28^ Two experts scored artifact-free data per AASM criteria^88^ in 20-s epochs.^28^

#### fMRI preprocessing

The fMRI data were preprocessed using HALFpipe (Harmonized AnaLysis of Functional MRI pipeline) version 1.2.2,^89^ incorporating fMRIprep.^90^ The first four volumes were discarded to reduce equilibrium effects. T1-weighted (T1w) images were corrected for intensity non-uniformity, segmented (CSF, white matter, gray matter), and normalized to MNI space via nonlinear registration. Functional data underwent slice-timing correction, motion correction, coregistration to T1w images, normalization to MNI space, and 8 mm FWHM smoothing.

### Quantification and statistical analysis

#### Event-related fMRI analyses

The fMRI data were analyzed using SPM12. Event-related analyses involved subject-level General Linear Models (GLMs). We computed four versions: the main model, a synchronization efficiency model, a sleep stage model, and a sleep stage × synchronization interaction model.

The main model included SWEs as events of interest convolved with the canonical hemodynamic response function (HRF), sleep spindles as regressors of no-interest, 24 motion parameters (six base head motion parameters, their temporal derivatives, and quadratic terms),^24^^,^^25^ five physiological noise regressors from CSF termed as anatomical component correction (aCompCor)^26^ and a constant term. Nuisance regressors were identical across all GLMs. Analyses were restricted to gray matter using a spatial mask (*p* > 0.20 from SPM12 tissue probability maps). For each subject, T-contrasts were used to examine the main effect of SWEs relative to baseline. Results were considered significant at *q* < 0.05_*FDRcorr*_ with a cluster extent threshold of *k* = 10 voxels.

In addition to the SWE regressor, the synchronization efficiency model included a parametric modulation regressor that contained synchronization scores for each individual SWE represented in the event regressor. Both regressors were convolved with the canonical HRF. Here, using *T*-contrasts, variations in the BOLD responses corresponding to the height of the SWEs’ synchronization score were examined. To test whether the spatial patterns associated with high versus low synchronization SWEs were influenced by SWE–spindle coupling, we conducted a follow-up analysis in which the SWE event regressor was paired with two parametric modulators: a binary coupling regressor (coupled = 1, uncoupled = 0) entered first, followed by the synchronization scores. Serial orthogonalization was applied to give coupling priority, allowing us to assess whether synchronization scores explained additional BOLD variance beyond that accounted for by coupling.

The sleep stage model included separate regressors for N2 and N3 SWEs, convolved with the canonical HRF. We contrasted the two to assess whether SWEs during N2 evoked different responses than during N3, and vice versa.

The interaction model included N2 and N3 SWE regressors along with their respective synchronization scores as parametric modulators. For each stage, we tested how BOLD responses varied with respect to the synchronization scores.

For the main and synchronization efficiency models, we generated probability maps of SWE-related activations. This approach is conceptually equivalent to penetrance maps widely used in fMRI to quantify interindividual consistency and variability of activation patterns.^20^^,^^21^^,^^22^^,^^23^ Specifically, subject-level T-maps were thresholded (*q* < 0.05_*FDRcorr*_, *k* = 10) and binarized (value of 1 for significant voxels, 0 otherwise). These individual binary maps were summed across all subjects within each dataset (*N* = 11 for dataset 1; *N* = 13 for dataset 2) and the resulting sum was divided by the respective *N* to compute a voxel-wise probability of activation. To define regions consistently recruited during SWEs, these probability maps were thresholded at a probability of ≥20% to create final reference masks. This threshold was selected to balance sensitivity to individual variability (e.g., differences in SWA expression and cross-anatomical variability) with adequate specificity.

To assess SWE-related BOLD activation in specific regions, we used a predefined atlas combining the Harvard-Oxford^91^ and AAL^92^ atlases. Regions of interest (ROIs) were defined, and the number of activated voxels per ROI and subject was extracted.
