# Supplementary material for: Slow wave canonical activity deviation concept: Toward a slow wave-based EEG-fMRI reference map for health-associated network function
Source: iScience. 2026 Mar 21;29(4):115455. doi: 10.1016/j.isci.2026.115455 (PMC13089066; doi:10.1016/j.isci.2026.115455)
Supplement: Document S1. Figures S1–S5 [file mmc1.pdf]

iScience, Volume *29*

Supplemental information

Slow wave canonical activity deviation concept: Toward a slow wave-based EEG-fMRI reference map for health-associated network function

Merve Ilhan-Bayrakcı, Oliver Tüscher, and Albrecht Stroh

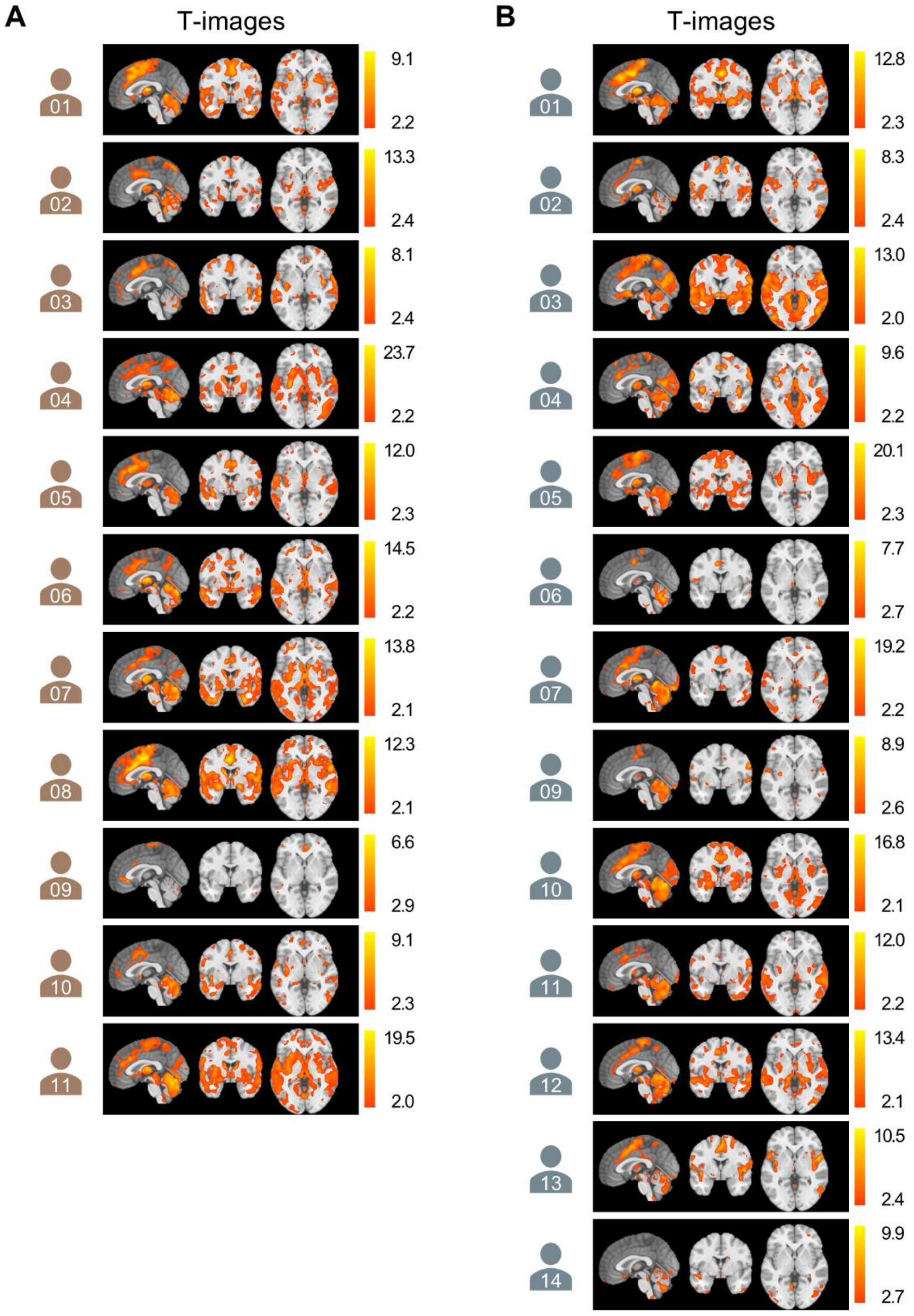

Figure S1. Individual SWE-related BOLD activation patterns for all subjects across both datasets, related to Figure 1.(A) Individual SWE-related BOLD fMRI statistical activation maps (T-images) for subjects from dataset 1 [S1]. The maps are thresholded at $q < 0.05$, FDR-corrected, with a cluster extent threshold of k = 10 voxels. (B) The same depiction for dataset 2 [S2]. Icons representing individual subjects are color-coded: brown for dataset 1 [S1] and grey for dataset 2 [S2].

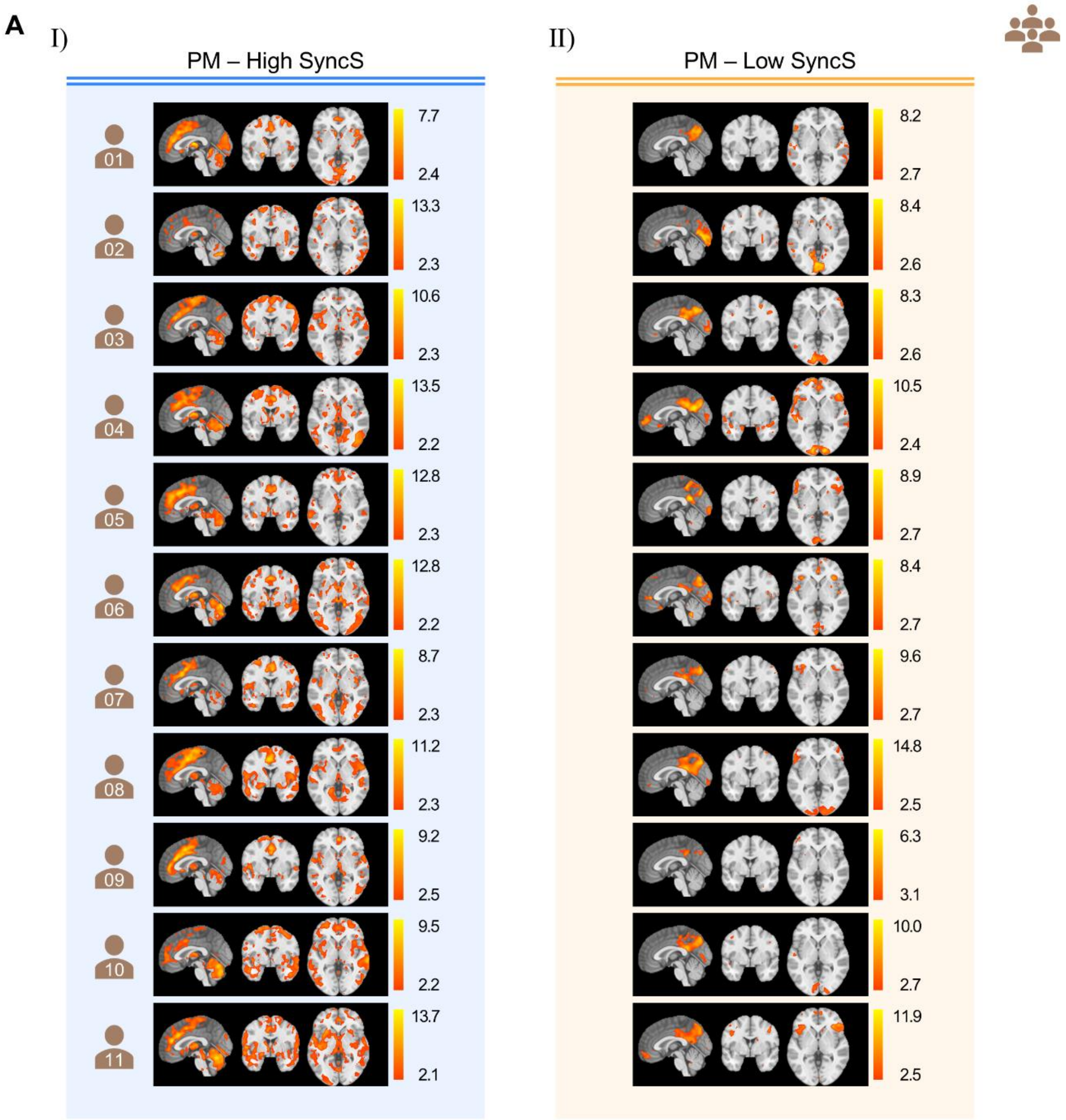

Figure S2. Distinct BOLD patterns associated with high vs. low synchronization efficiency of SWEs (dataset 1), related to Figure 2. (A) Ⅰ) BOLD response patterns of individual subjects indicating brain regions where the BOLD signal increases with higher synchronization scores (SyncS) *(q* < 0.05$_{FDRcorr,}$ cluster extent threshold: *k* = 10 voxels*)*. Ⅱ) BOLD response patterns of individual subjects showing brain regions where the BOLD signal increases with lower synchronization scores *(q* < 0.05$_{FDRcorr,}$ cluster extent threshold: *k* = 10 voxels*)*. Brown icons represent individual subjects. The icon in the upper right corner designates the dataset, with the brown group symbol representing dataset 1 [S1].

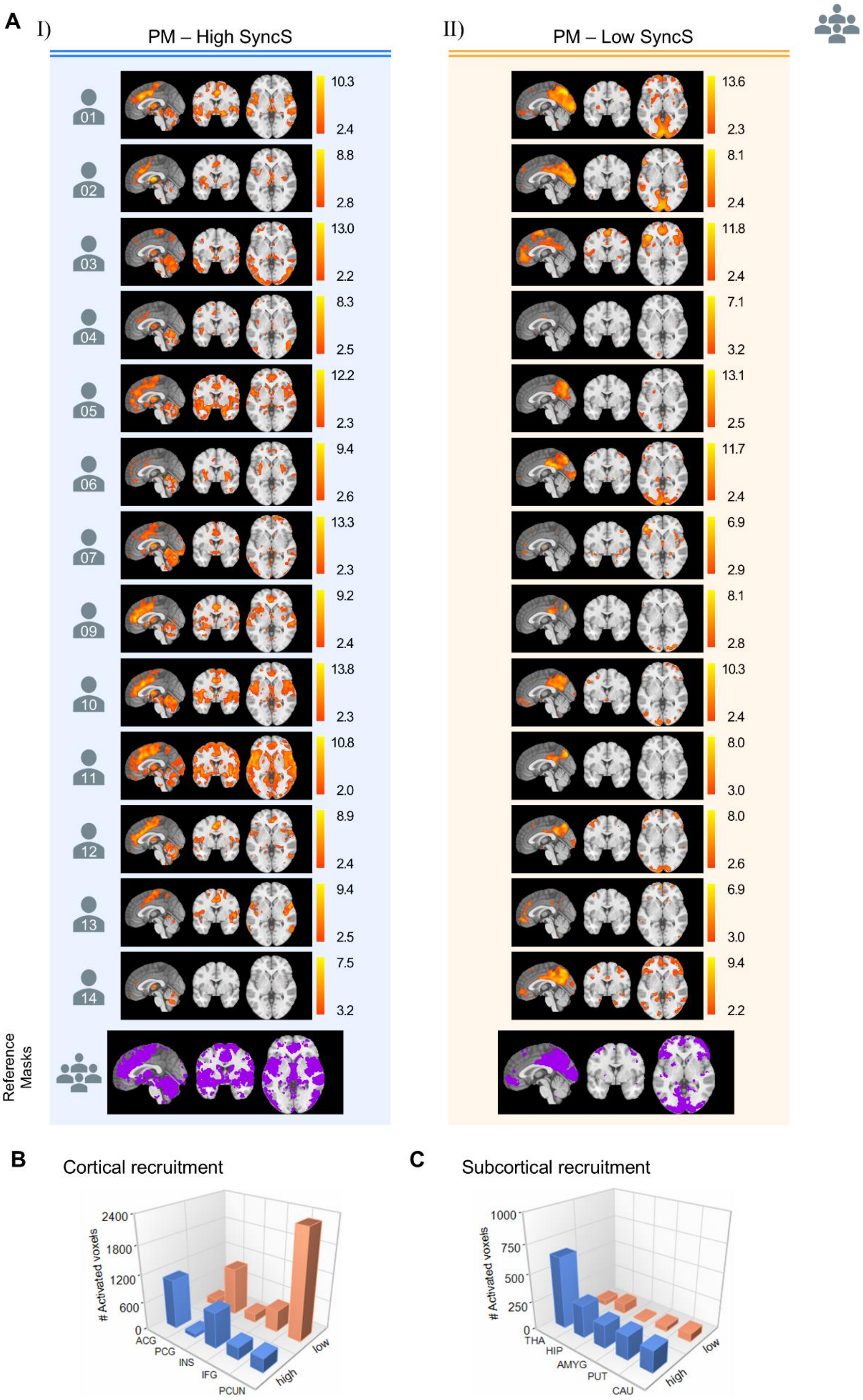

Figure S3. Distinct BOLD patterns associated with high vs. low synchronization efficiency of SWEs (dataset 2), related to Figure 2. (A) Ⅰ) BOLD response patterns of individual subjects indicating brain regions where the BOLD signal increases with higher synchronization scores (SyncS) *(*$q < 0.05_{FDRcorr}$*,* cluster extent threshold: k = 10 voxels*)*, along with the corresponding spatial reference mask. Ⅱ) BOLD response patterns of individual subjects showing brain regions where the BOLD signal increases with lower synchronization scores *(*$q < 0.05_{FDRcorr}$*,* cluster extent threshold: k = 10 voxels*)*, along with the corresponding spatial reference mask. Grey icons represent individual subjects. (B) A 3D bar plot shows the mean number of activated voxels across individual subjects in cortical regions for both SWEs with high and low synchronization scores, represented in blue and orange, respectively. Abbreviations: ACG, anterior cingulate gyrus; PCG, posterior cingulate gyrus; INS, insula; IFG, inferior frontal gyrus; PCUN, precuneus. (C) Mean voxel recruitment in subcortical areas across individual subjects for both SWEs with high and low synchronization scores depicted in a 3D bar plot. Abbreviations: THA, thalamus; HIP, hippocampus; AMYG, amygdala; PUT, putamen; CAU, caudate. Upper-right icon designates the dataset, with the grey group symbol representing dataset 2 [S2].

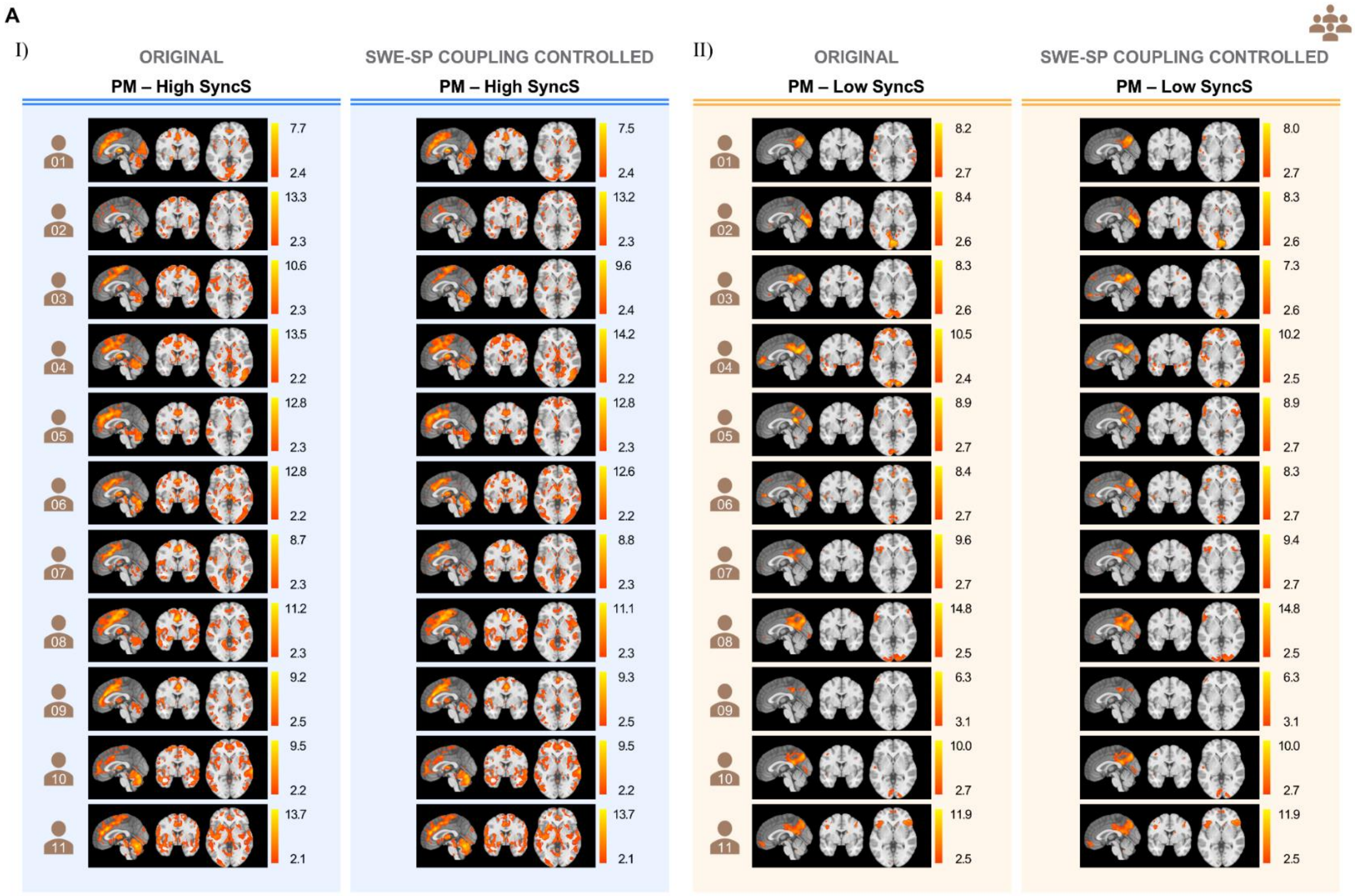

Figure S4. Comparison of original and SWE-spindle (SWE-SP) coupling-controlled BOLD response patterns associated with synchronization scores (SyncS) (dataset 1). (A) Ⅰ) High synchronization SWEs: Left column shows BOLD response patterns indicating regions where the BOLD signal increases with higher SyncS in the original analysis. Right column shows the corresponding patterns after controlling for SWE–spindle coupling *(*$q < 0.05_{FDRcorr}$*,* cluster extent threshold: k = 10 voxels*)*. Ⅱ) Low synchronization SWEs: Left column shows BOLD response patterns where the BOLD signal increases with lower SyncS in the original analysis. Right column shows the SWE-spindle coupling-controlled patterns for low SyncS. *(*$q < 0.05_{FDRcorr}$*,* cluster extent threshold: k = 10 voxels*)*. Brown icons represent individual subjects. The icon in the upper right corner designates the dataset, with the brown group symbol representing dataset 1 [S1].

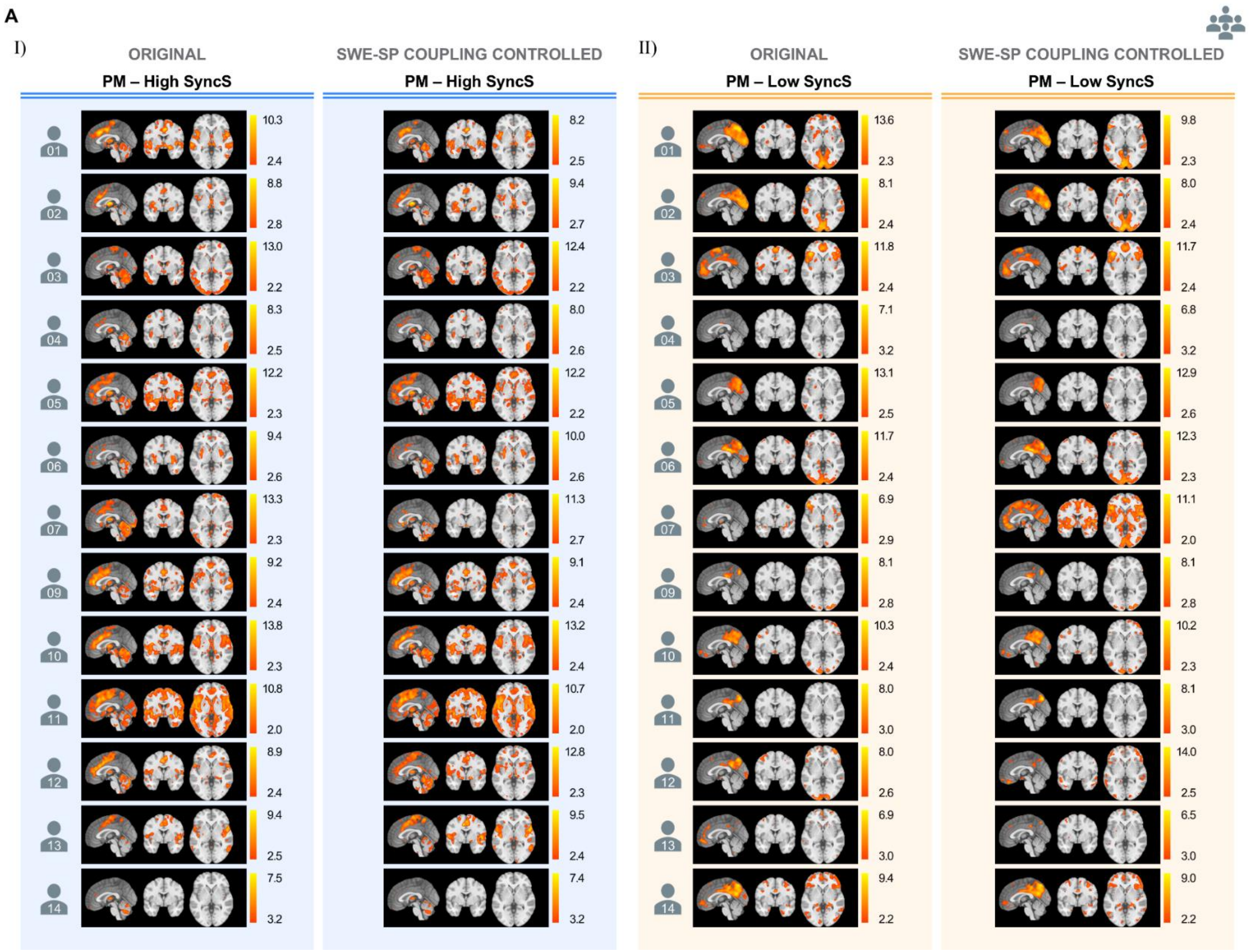

Figure S5. Comparison of original and SWE-spindle (SWE-SP) coupling-controlled BOLD response patterns associated with synchronization scores (SyncS) (dataset 2). (A) Ⅰ) High synchronization SWEs: Left column shows BOLD response patterns indicating regions where the BOLD signal increases with higher SyncS in the original analysis. Right column shows the corresponding patterns after controlling for SWE–spindle coupling *(q < 0.05$_{FDRcorr}$,* cluster extent threshold: k = 10 voxels*)*. Ⅱ) Low synchronization SWEs: Left column shows BOLD response patterns where the BOLD signal increases with lower SyncS in the original analysis. Right column shows the SWE-spindle coupling-controlled patterns for low SyncS. *(q < 0.05$_{FDRcorr}$,* cluster extent threshold: k = 10 voxels*)*. Grey icons represent individual subjects. The icon in the upper right corner designates the dataset, with the grey group symbol representing dataset 2 [S2].

Supplemental References

[S1] Bergmann, T.O., Mölle, M., Diedrichs, J., Born, J., and Siebner, H.R. (2012). Sleep spindle-related reactivation of category-specific cortical regions after learning face-scene associations. NeuroImage 59, 2733–2742. https://doi.org/10.1016/j.neuroimage.2011.10.036.

[S2] Sterpenich, V., van Schie, M.K., Catsiyannis, M., Ramyead, A., Perrig, S., Yang, H.-D., Van De Ville, D., and Schwartz, S. (2021). Reward biases spontaneous neural reactivation during sleep. (OpenNeuro). https://doi.org/10.18112/openneuro.ds003574.v1.0.2.
